# A rare case of a composite phaeochromocytoma-ganglioneuroma in Australia

**DOI:** 10.1093/jscr/rjaf184

**Published:** 2025-04-03

**Authors:** Peter Pham, Benedict Kakala, Amanda Love, Archana M Sudarsan, Clement Wong

**Affiliations:** Breast & Endocrine Surgical Department, Royal Brisbane and Women’s Hospital, Herston, QLD 4006, Australia; Breast & Endocrine Surgical Department, Royal Brisbane and Women’s Hospital, Herston, QLD 4006, Australia; Endocrinology Department, Royal Brisbane and Women’s Hospital, Herston, QLD 4006, Australia; Pathology Department, Royal Brisbane and Women’s Hospital, Herston, QLD 4006, Australia; Breast & Endocrine Surgical Department, Royal Brisbane and Women’s Hospital, Herston, QLD 4006, Australia; The University of Queensland, Brisbane, 4006, Australia

**Keywords:** composite, phaeochromocytoma, ganglioneuroma, surgical

## Abstract

Composite phaeochromocytomas (CP) of the adrenal medulla are rare neuroendocrine tumours, comprising phaeochromocytoma and neurogenic components. They may present heterogeneously like ordinary phaeochromocytomas and are ideally managed surgically with a multidisciplinary approach. This report describes the case of a CP in Australia, which was cured by a surgical team in an unplanned two-staged left adrenalectomy over a 20-year period.

## Introduction

Composite phaeochromocytomas (CP) are rare neuroendocrine tumours characterized by coexistence of phaeochromocytoma and neurogenic components. The neurogenic component includes ganglioneuroma, ganglioneuroblastoma, neuroblastoma, schwannomas, and peripheral nerve sheath tumours [1]. Our case, of a composite phaeochromocytoma-ganglioneuroma, represents the most reported phenotype of CP, seen in ⁓75% of all CP cases [1].

The aetiology of CP is related to the common embryologic origin of its tumour components [2]. The chromaffin cells of phaeochromocytomas and ganglion cells of ganglioneuromas are both derived from neural crest cells, which, during embryologic life, differentiate to give rise to the cellular components of the adrenal medulla and sympathetic ganglia [2]. Any divergence in this differentiation process can lead to the development of composite tumours.

Clinically, phaeochromocytoma-ganglioneuroma composites are managed like ordinary phaeochromocytomas. Surgical resection is the main curative method, with a multidisciplinary approach involving endocrinologists, anaesthetists, and surgeons [3].

We present the case of a 79-year-old woman, with a composite phaeochromocytoma-ganglioneuroma, who was cured by an Australian surgical team in an unplanned two-staged left adrenalectomy over a 20-year period.

## Case report

**Figure 1:**
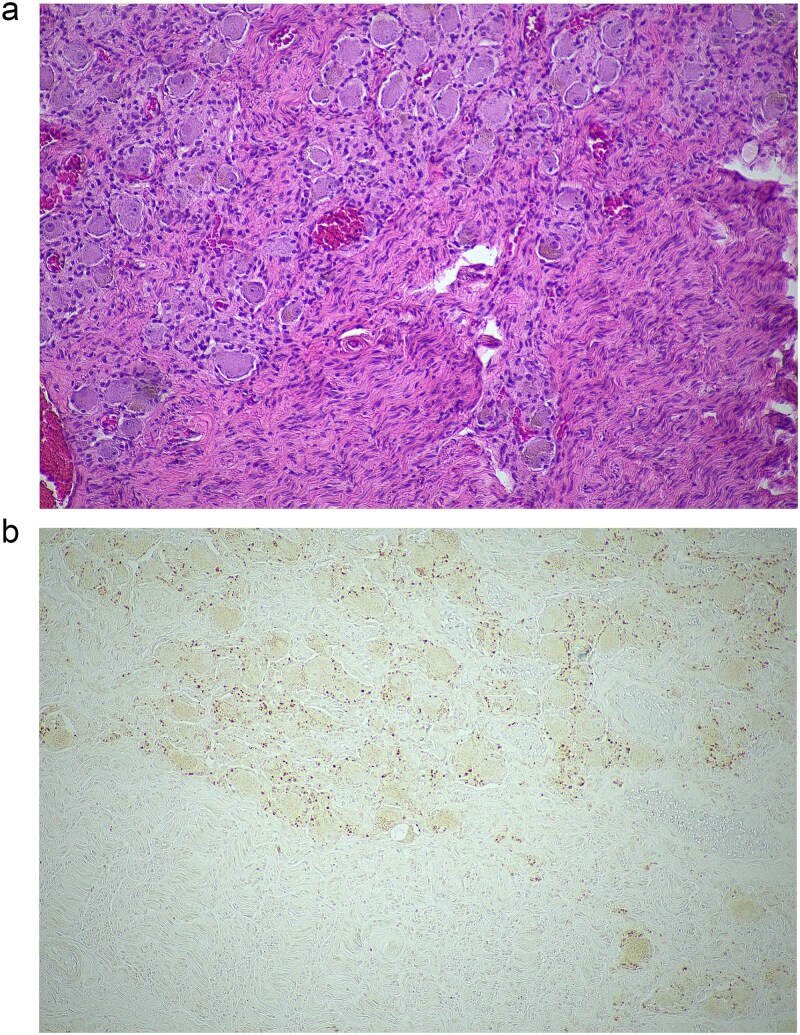
Histopathological examination of the excised lesion—from the first stage partial adrenalectomy—demonstrates characteristic features of a ganglioneuroma. (a) The section shows mature neural tissue with ganglion cells, with all neural tissue lighting with S100 (haematoxylin and eosin staining; 10× magnification). (b) The neoplastic cells are negative for chromogranin (10× magnification).

A 79-year-old female was referred to Endocrinology in 2023 with a 2.6 cm left adrenal nodule suspicious for a phaeochromocytoma. She had a laparoscopic partial left adrenalectomy in 2004 for a left adrenal nodule concerning for lung squamous cell carcinoma (SCC) oligometastasis. The 2004 surgery was complicated by acute pulmonary oedema and severe cardiomyopathy with an ejection fraction (EF) of 10%. She underwent emergency coronary angiography (normal) and an intra-aortic pump was placed. The patient’s cardiac function quickly recovered with an EF 50% after one week. The Intensivist and Cardiologist diagnosed catecholamine surge as the most likely cause. She was supported haemodynamically with an intra-aortic balloon pump rather than with inotropes. There was no biochemical evaluation for catecholamine excess prior to this surgery. Histology was consistent with a ganglioneuroma (no evidence of metastatic lung cancer or phaeochromocytoma) (Fig. 1).

The patient had other surgeries including a right lower lobectomy for a SCC, total abdominal hysterectomy with bilateral salpingo-oophorectomy for multiple leiomyomata, and endoscopic resection of a low-grade gastric gastrointestinal stromal tumour. She has no family history of endocrine tumours or familial cancer syndromes.

On review by Endocrinology in 2023, the patient reported frequent headaches, nocturnal sweating, and palpitations. On physical examination, she was hypertensive (170/106 mmHg) with a normal heart rate. Abdominal examination for masses or lymphadenopathy was unremarkable.

Initial biochemical evaluation revealed an elevated plasma normetadrenaline 2970 (<1560) and metadrenaline 1280 (<447), with normal 3-methoxytyramine. Aldosterone: renin ratio and 1 mg dexamethasone suppression test were normal.

**Figure 2:**
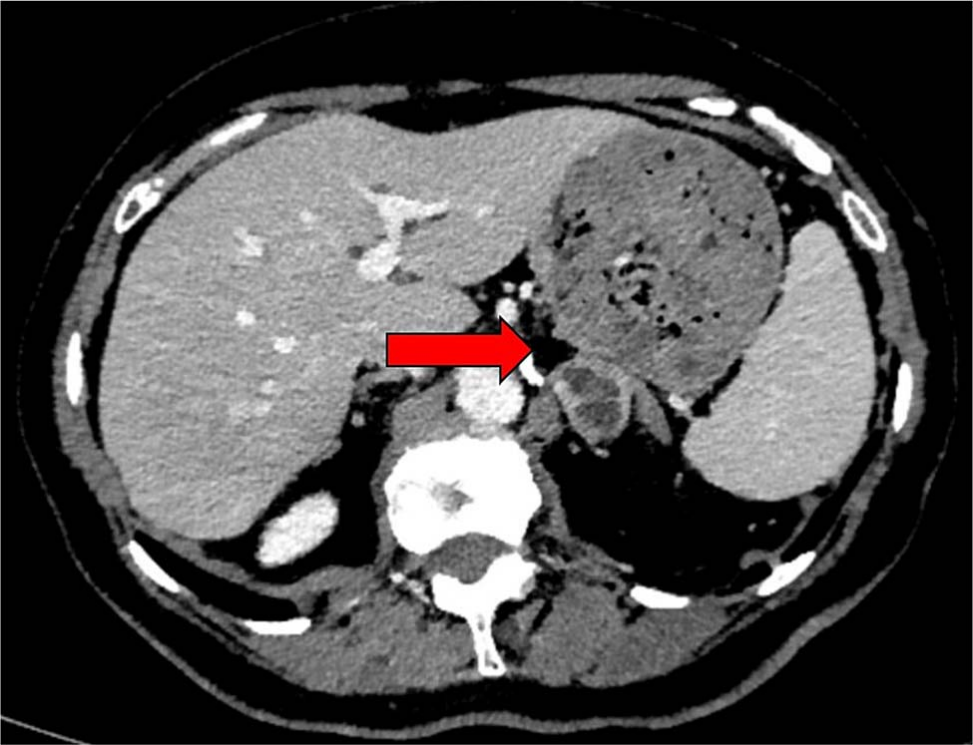
Preoperative axial CT image in 2024 showing an indeterminate left adrenal lesion measuring 28 × 26 × 13 mm, with heterogenous postcontrast enhancement and internal cystic change (indicated by arrow). Absolute and relative washout were calculated at 48.3% and 23.3%, respectively.

Computed tomography (CT) adrenal demonstrated a 2.8 × 2.6 cm left adrenal lesion (Fig. 2) that had increased in size when compared with the CT of 18 months prior (2.6 × 2.5 cm). The CT findings, combined with the clinical presentation and elevated plasma metanephrines, raised suspicion for a left phaeochromocytoma.

**Figure 3:**
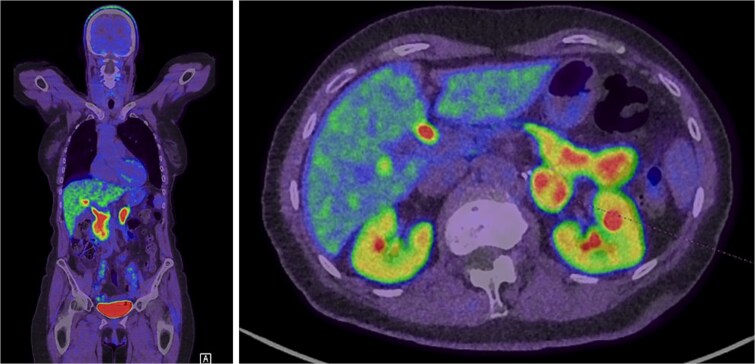
Intense FDOPA uptake is demonstrated in the left adrenal lesion, which is in keeping with a catecholamine-secreting tumour.

Further evaluation was done to exclude paragangliomas and distant metastases. F-Fluorodopa (FDOPA) positron emission tomography (PET) was performed and showed strong avidity in the lesion suggesting a catecholamine-producing tumour (Fig. 3). She was referred for consideration of a left adrenalectomy. Preoperative preparation with alpha-blockade (Prazosin) was commenced and 17 gene panel testing was negative for a pathogenic variant.

**Figure 4:**
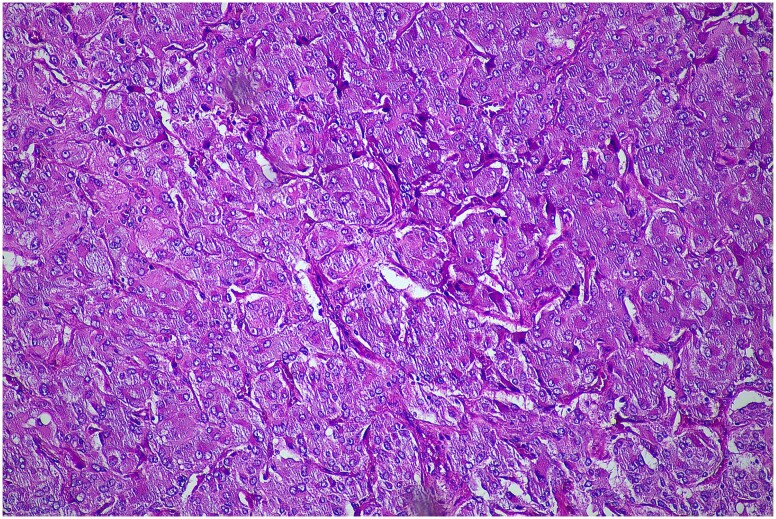
Histopathological examination of the excised lesion—from the second stage completion adrenalectomy—demonstrates characteristic features of a phaeochromocytoma. The section shows atypical polygonal cells with large hyperchrome nuclei, and cytoplasm with a granular appearance S100 (haematoxylin and eosin staining; 10× magnification). The cells are arranged in trabeculae and surrounded with s100 positive supporting cells.

In February 2024, the patient underwent a left re-do retroperitoneoscopic completion adrenalectomy. Her postoperative course was uncomplicated, and she was discharged home within two days. The histopathology confirmed a low-risk phaeochromocytoma (Fig. 4). The plasma metanephrines normalized.

The two adrenal operative specimens, from the first stage partial adrenalectomy (2004) and second stage completion adrenalectomy (2024), were reviewed at our Multidisciplinary Team meeting. The histology was unequivocally different on the two occasions, with no phaeochromocytoma tissue evident on the first surgical specimen, and no residual ganglioneuroma evident on the second specimen. Given transformation of a ganglioneuroma into a pheochromocytoma is very unlikely, we postulate that, based on her complications during the first surgery, she must have had a phaeochromocytoma present—in addition to the ganglioneuroma—which was left behind and has grown over the intervening years. It was interesting that in the 20 years between the two operations she did not suffer another catecholamine crisis.

## Discussion

‘CP’ is used to describe a phaeochromocytoma in combination with a non-phaeochromocytoma component, whereby the additional component arises from a common embryonic progenitor (i.e. neural crest) [4]. In our case, the non-phaeochromocytoma component was a ganglioneuroma.

There are ⁓110 cases of CP published in the literature to date [5]. They constitute 1%–9% of all phaeochromocytomas and <3% of all sympathoadrenal tumours [1, 6]. The median age at diagnosis is 51.5 years, with the majority presenting between the fourth and sixth decades [5]. Our patient had her index operation at 59 years.

Not only does the low incidence of CP make them difficult to detect, but CP may manifest similarly to pure phaeochromocytomas [7]. The symptomatology of CP is highly heterogenous and is related to the functionality of either component of the tumour [1]. Patients may present with symptoms of catecholaminergic excess, including the classic triad of headache, sweating, and palpitations [5]. Approximately 25% of CP are metabolically inactive and are asymptomatic [8].

Radiographically, CP are indistinguishable from pure phaeochromocytomas. As observed in the present case, ganglioneuromas and phaeochromocytomas appear similarly as well-circumscribed, heterogenous, or cystic adrenal masses with or without calcification [9]. There is no structural imaging modality that can accurately differentiate ganglioneuromas from phaeochromocytomas. Functional imaging with F-DOPA can detect catecholamine producing tumours [10].

Definitive diagnosis of CP requires histopathological examination [1, 11]. Microscopically, the components of CP typically resemble their counterparts in pure tumours of the same type. Phaeochromocytomas are often composed of polygonal or spindle-shaped cells arranged in a nested (Zellballen), trabecular or solid pattern, with granular cytoplasm and round to oval nuclei [7]. The ganglioneuroma component, on the other hand, is composed of Schwann cells and ganglion cells, and stains strongly for the S100 and neurofilament biomarkers [12]. Importantly, scattered ganglion cells and Schwannian-like stroma can be seen in pure phaeochromocytomas, and so their mere presence is not sufficient to warrant diagnosis of a CP [4]. The World Health Organization diagnostic criterion suggests that at least 5% of each component should comprise a CP to qualify classification [13]. The two components of the tumour may be admixed or separate.

In our case, microscopic examination of the surgical specimens demonstrated morphologic compatibility with a ganglioneuroma and phaeochromocytoma. The two components were separate; but uniquely, presented as distinct pathologies in different specimens. The CP with separate tumour entities is very rare compared to the admixed subtype.

Overall, the prognosis of CP is good. These tumours are typically benign, with patients generally having favourable clinical outcomes following surgical resection. Costa *et al.* reported that the rate of metastatic disease of CP is 8.2% [5].

We examined the epidemiological, clinicopathological, and prognostic features of CP. Our case required curative surgical resection 20 years after the initial surgery, which appears to be the first case of its kind. The identification of different histological types of tumours, in separate adrenal specimens, added complexity to reaching a final diagnosis. Clinicians should maintain a high index of suspicion and order biochemical screening for this condition, as more research is vital to fully understand its behaviour and outcomes.
